# Preferences for Attachment Devices for Individuals with Lower-Limb Loss: A Discrete-Choice Study to Inform Regulatory Decisions

**DOI:** 10.1177/23814683251351044

**Published:** 2025-07-02

**Authors:** Leslie Wilson, Matthew Garibaldi, Ruben Vargas, Molly Timmerman

**Affiliations:** Department of Clinical Pharmacy, University of California San Francisco, San Francisco, CA, USA; Orthotics & Prosthetics, University of California, San Francisco, San Francisco, CA, USA; Department of Clinical Pharmacy, University of California San Francisco, San Francisco, CA, USA; General Physical Medicine & Rehabilitation, VA Palo Alto Health Care System, Palo Alto, CA, USA

**Keywords:** discrete choice, osseointegration, chronic low back pain, conjoint analysis, risks, benefits, FDA

## Abstract

**Objective.** The patient’s perspective in shared decision making has expanded to regulatory decision making for medical devices under the Food and Drug Administration’s Patient Preference Initiative. **Methods.** Using choice-based conjoint (CBC) procedures, a discrete-choice experiment measure describing the risks and benefits of osseointegration was designed and used in a preference study among 188 adults with lower-limb loss. Our measure included 8 attributes of 1) risks: chance of infection, complete device failure rate, time without prosthesis, activity limitations, and 2) benefits: avoidance of socket problems, limb perception, improved motion with less fatigue, and chance of limiting daily pain, with 3 to 4 levels each. We used a random, full-profile, balanced-overlap design in which 18 CBC conjoint pairs, sociodemographic, and clinical questions were completed. The analysis included random parameters logit with 1,000 Halton draws and latent class. **Results.** The least important levels, when compared with their respective attribute baseline level, were for avoiding the highest chance (50%) of serious infection (β = −1.32, *P* < 0.001), highest chance (40%) of complete device failure (β = −0.96, *P* < 0.001), and longest (9 mo) time without prosthesis (β = −1.12, *P* < 0.001). The most preferred levels, when compared with their respective attribute baseline level, were to eliminate daily pain (β = 0.87, *P* < 0.001; β = 0.62, *P* < 0.001). The preference for avoiding current osseointegration infection risk (10%) was much lower (β = 0.51, *P* < 0.001), showing that preferences to avoid the actual infection risk are offset by osseointegrations benefits. Latent class analysis showed 2 distinct classes with some risk averse and some with more balanced preferences. **Conclusions.** The strongest preferences were seen for attributes avoiding complications; however, individuals demonstrated a willingness to make risk–benefit tradeoffs at current risk levels. These findings can guide future regulatory prosthetic decisions and allow better shared decision making to decrease prosthetic abandonment.

**Highlights:**

- Importance shown to avoid the actual infection risk of osseointegration can be offset by the individual’s importance for the benefits of osseointegration to avoid pain and socket problems and to have rapid device snap on.
- Individuals also showed they are willing to trade the actual osseointegration device failure rate risks for the likely benefits of osseointegration.
- Individuals strongly preferred avoiding time without the use of a prosthetic for the time it takes to undergo and recover from the osseointegration procedure, informing the debate for favoring a faster procedure and recovery time if it is safe. However, these preferences were still in the tradeoff range for the benefits of osseointegration.
- Individuals showed the strongest importance for a potential benefit of osseointegration to reduce the presence of daily pain, which is a likely benefit of the osseointegration procedure, especially when accompanied by targeted muscle reinnervation.
- We showed considerable heterogeneity across patient preferences, with one group showing a strong importance for avoiding risks but also importance for some benefits of osseointegration and a larger group who showed a more moderate importance across both risks and benefits.

The patient’s perspective in shared decision making has expanded to regulatory decision making for medical devices under the Food and Drug Administration’s (FDA’s) Patient Preference Initiative. Several initiatives have been launched to expand patient preference information within the FDA. First, the 5 Centers of Excellence in Regulatory Science and Innovation, whose goals are to advance regulatory science, are collaborating around the methodological development, education, and dissemination of patient preference information with the FDA. The Medical Device Innovation Consortium published the first framework suggesting the use of discrete choice for measurement of patient preference for integration into regulatory benefit–risk decisions and the Society for Medical Decision Making and ISPOR interest groups described the conceptual methods.^1^ Then, the FDA (Center for Devices and Radiological Health; CDRH) developed a guidance for submission of patient preference information for regulatory decisions^2^ and funded case studies to test the use of patient preference information for what they define as “preference-sensitive conditions” needing regulatory decisions across the FDA.^3–6^

The rapid development of prosthetic devices seeking FDA approval, along with the knowledge that 22% of individuals with lower-limb loss (LLL) abandon their device, put prosthetic device advances on the CDRH list of patient-sensitive conditions and supplied funding for this preference study and encouraged a Q-submission with feedback.^7,8^

Our objective is to describe the development of a discrete-choice measure for measuring patient preference for a new device attachment procedure (osseointegration) and its use in a population of those with LLL to determine how patients weigh the risks and benefits of an osseointegration attachment to better inform regulatory and clinical decision making.^9^

## Discrete Choice

Various methods are available to measure patient preference, with discrete choice favored for its ability to quantitatively measure individuals’ tradeoffs. Discrete-choice experiments (DCEs) require identification of attributes crucial for decision making and their defined levels describing their range. Individuals are then presented with multiple choice sets, each featuring different levels of the same attributes, and asked to choose which they prefer. Their repeated choices are quantified into utility scores (β coefficients from regression analysis). These utility scores indicate both the strength of preference (importance) for each attribute level and the willingness to exchange levels of risk and benefit during decision making.

## Osseointegration Attachment Innovation

Osseointegration involves surgical implantation of a titanium post into bone, which is anchored by the growth of bone and tissue around it. This osseointegrated attachment device allows easy snap on of any prosthetic device and eliminates skin problems from socket attachment.^10^ Because the post protrudes from the skin, there is a relatively small, but constant, infection potential and also a risk of bone fracture if a severe fall had affected that limb. Osseointegration requires 1 to 2 surgical procedures and rehabilitation time to build up leg strength, which delays full prosthetic use for up to 6 mo.^11^ These specific risks and benefits make this device preference sensitive for the individual, making it essential to determine how individuals make these tradeoffs when choosing osseointegration to inform the FDA of their perspective.

## Methods

### Study Design

This is an observational study of the development and pilot testing of a discrete-choice measure and an observational assessment of patient preference for the risks and benefits of osseointegration in individuals with LLL. The study was approved by the University of California San Francisco (UCSF) Institutional Review Board (IRB).

### Instrument Development

The discrete-choice measure was developed following the guidelines for attribute development, including a literature review of the risks and benefits of osseointegration, 10 one-on-one interviews with individuals with limb loss and 3 prosthetist experts, and 4 to 5 individuals from the FDA responsible for reviewing prosthetics for approval who all made survey changes.^1,12,13^ We also formally presented the measure to an FDA panel responsible for approving prosthetic devices through the Q-submission process. The final CBC instrument was then pilot tested in a sample of 64 adults with LLL, before combining with a larger implementation sample.

### Recruitment and Inclusion Criteria

Recruitment was from UCSF clinics, the Amputee Coalition advocacy group, Hanger Clinics, the Stanford Veterans Affairs (VA) Medical Center, and a limb-loss Reddit interest group. Inclusion criteria were adults aged 18+ y with above-the-ankle LLL and could be those with or without an osseointegration device. One fixed-choice question showed all the best choice levels versus all the worst choice levels in the choice pair to discern whether individuals were genuinely attentive and understood the survey. Those choosing all the worst attribute levels and incomplete responses were excluded from the analysis.

### Measure Design and Data Collection

The selection of our DCE choice-based conjoint (CBC) experimental-design algorithm was made with Sawtooth Software (Sawtooth Software, Inc., Provo, UT, USA), where a random, full-profile, balanced overlap design was applied to maximize the information acquisition for each response. Sawtooth’s Web-based administration method was used to deliver the survey. The final CBC included 18 random and 2 fixed-task pairs. The pilot study and full baseline study use the same attributes and design. Images were used to demonstrate the characteristics of the selected attribute levels to better engage respondents (Appendix Figure 1). A $10 payment was given upon completion of the survey.

### Other Measures

Demographics and questions about the cause, limb level, timing, and other characteristics of limb loss; prosthetic use; comfort/satisfaction history; and the Questionnaire for Persons with a Transfemoral Amputation (Q-TFA) were also gathered. The Q-TFA is a widely used measure with a prosthetic use score, a prosthetic mobility score, a prosthetic problems score, and a global prosthetic health score.^14,15^

### Statistical Analysis

Using R version 4.3.2 (2023-10-31), we report the descriptive statistics of the respondent’s demographic and LLL characteristics. The primary outcome is the CBC utility value associated with each attribute level, analyzed with the GMNL package to estimate random-parameter (mixed) logit models using dummy coding with 1,000 Halton draws. The respondent’s choice of each hypothetical prosthesis scenario pair is the dependent variable, while the attribute levels are the independent variables. The preference weights (β coefficients) are the unitless part-worth utility of each attribute level and indicate the relative importance of the risk or benefit to the individual. A larger preference weight suggests a stronger importance, with negative preference weights indicating importance to avoid that attribute level. Most of our analyses were done with dummy coding and categorical variables, so each attribute level is the utility preference relative to a zero base. Confidence intervals and significance values are reported. The risk variables were analyzed separately as both categorical and continuous variables. For analysis with continuous variables, the β coefficients indicate the preference strength with each 1% increase or decrease in that attribute level. We additionally analyzed by selected subgroups. To address the possibility of scale differences across subgroups, we used the likelihood ratio test (LRT) to compare 6 individual random parameter logistic regression (RPL) preference models (heteroskedastic) using each subgroup variable as the scale parameter, with the overall base RPL preference model. A significant result indicates the possibility of a scale difference for that subgroup variable.^16^

Further analysis was conducted using RPL with interaction terms by different subsets of variables that might influence preferences for osseointegration (males/females, unilateral v. bilateral limb loss, age group, low/high ease of use). In addition, latent class analysis was conducted to identify underlying groups within the sample with similar preferences both without and with these variables used as covariates, aiding in the understanding of heterogeneity within the population.

An attribute importance score was also calculated using effects coding and categorical variables. For each attribute, we subtracted the highest and lowest β coefficient for the levels in that attribute. These scores were then standardized to a scale of 1 relative to the most important attribute to determine the attribute-specific relative importance score. To normalize, we used the following formula: Normalized Importance Score_Attribute2_ = 1 × (Attribute2 . . . 7_highest level_– Attribute2 . . . 7_lowest level_)/(Most Imp Attribute_highest level_– Most Imp Attribute_lowest level_).^17,18^ We also compared all attributes of benefit against the highest risk attribute and all attributes of risk against the highest benefit attribute, using the same calculation. This allows a comparison of how much risk individuals are willing to trade for a specific benefit and vice versa. We first calculated the maximum acceptable risk (MAR) score of 2 of the important risk attributes in relationship to the selected benefit level difference and standardized to 10 with the following formula: MAR = 100 × (Benefit 1 . . . 7_highest level_– Benefit1…7_lowest level_)/(Most Imp Risk Attribute_highest level_– Most Imp Risk Atribute_lowestlevel_). We then used the Simultaneous Maximum Acceptable Risk Threshold (SMART) approach to consider the simultaneous importance of selected risks and benefits.^19–21^ Assuming the linearity of our risks, we mapped the individual MARs for both of our major risks (serious infection and complete device failure) as *x* and *y* intercepts connected by a line for each of the major benefits selected to show the combination risk threshold for each benefit change compared. To better understand the effect on the overall CBC results of respondents with low data quality, we used STAT garbage class MIXL models.^22–24^ First, we fit a standard panel MIXL model, which presents posterior summary statistics for the MIXL mean estimates, while the (co)variance estimates were separated into standard deviation (SD) and correlation estimates. We then fit the garbage class MIXL model to provide the MIXL estimates that are corrected for the presence of respondents with poor-quality data. This will estimate the size of the garbage class in our dataset.^22–24^

## Results

### Instrument Description

The final measure included 8 attributes, with 3 to 4 levels each describing a broad but plausible range of the risks and benefits of osseointegration (Appendix Figure 1, Table 1). Attributes of risk included 1) the chance of serious but hospital-treatable infection; 2) the chance of complete device failure for 5 y; 3) total time without prosthesis for surgery, rehabilitation, or training; and 4) limitations on activities with and without a prosthetic. The attributes of benefit included 1) avoidance of socket problems, 2) ability to sense limb perception, 3) improved joint range and gait of motion with less fatigue, and 4) avoidance of chance of daily pain (Table 1, Appendix Figure 1).

**Table 1:** Additional Information for Reference Only: This Is an Example of the Features and Their Definitions That We Will Be Asking You about on the Survey in Case You Need It

**Instructions: LOWER LIMB LOSS AND LIMB DIFFERENCE** • There is a new ATTACHMENT DEVICE for a LOWER-LIMB prosthetic available with both good and bad features. • We will show you 2 choices of this ATTACHMENT DEVICE, 20 times, each with different features. • Please choose which prosthetic ATTACHMENT DEVICE you prefer (option 1 or option 2) each time. There is no right or wrong answer.				
Text on Online Survey			Additional Information	
	Features		Longer Description	Explanation and Example
1.	Chance of serious but hospital treatable infection is:	0 in 100	0 in 100 chance that you will have a serious infection. You may have local draining around the attachment device, but only daily cleaning is required.	Each presented option that you might choose may have a different chance for this serious infection. This infection can be treated with 3 days in the hospital and 2 weeks taking an infused drug.
		1 in 100	1 in 100 chance that you will have a serious infection but one that can be treated in the hospital and then 2 weeks at home with IV antibiotics.	
		10 in 100	10 in 100 chance that you will have a serious infection but one that can be treated with 3 days in the hospital and then 2 weeks at home with IV antibiotics.	
		50 in 100	50 in 100 chance that you will have a serious infection but one that can be treated with 3 days in the hospital and then 2 weeks at home with IV antibiotics.	
2.	Chance of complete device failure:	0 in 100	There is NO (0 in 100) chance that the prosthetic attachment device will fail because you continue to use a socket attached prosthetic.	Each presented option that you might choose may have a different chance for this complete device failure, resulting in complete removal of the device and need to heal before again using your socket prosthetic. This failure can be due to many things, including a broken bone, loosening of the prosthetic over time, skin dying around the prosthetic, a deep bone infection, or your inability to adapt to the prosthetic. If your prosthetic fails, you may not be able to use any other prosthetic for at least 3 years.
		1 in 100	There is 1 in 100 (1%) chance that the prosthetic attachment device will fail causing its removal and need to heal for at least 3 years before again using your socket prosthetic.	
		10 in 100	There is 10 in 100 (10%) chance that the attachment device will fail causing its removal and need to heal for at least 3 years before again using your socket prosthetic.	
		40 in 100	There is 40 in 100 (40%) chance that the prosthetic attachment device will fail causing its removal and need to heal for at least 3 years before again using your socket prosthetic.	
3.	Time without prosthetic for 2 surgeries, rehabilitation, and training:	No time without prosthetic, no surgeries/rehabilitation	There is no time needed without using your prosthetic, and no surgeries or rehabilitation are needed.	Each presented option that you might choose may have a different amount of time that you have no prosthetic weight bearing allowed while you have 2 surgical procedures to implant a titanium rod into your bone and as well as have weekly rehabilitation and training until full weight bearing is allowed.
		9 mo	This prosthetic attachment devices requires 9 months without prosthetic use, for 2 surgeries to implant the titanium rod into your bone, as well as weekly rehabilitation and training before full weight bearing.	
		4 mo	This prosthetic attachment devices requires 9 months without prosthetic use, for 2 surgeries to implant the titanium rod into your bone, as well as weekly rehabilitation and training before full weight bearing.	
4.	Activity limitations:	No limitations	There are no activity limitations with this attachment device.	Each presented option that you might choose may require different activity limitations when you use the prosthetic attachment device.
		No impact sports	It is suggested that you play no impact sports with this prosthetic attachment device.	
		No public pools	It is suggested that you do not swim in public pools with or without your prosthetic with this attachment device.	
		No excess pivot twists	It is suggested that you do not perform activities with excess pivot twists or risk of falls with this prosthetic.	
5.	Avoid socket problems:	No sensations	You have daily chafing, perspiration, and long donning time using your socket attachment.	Each presented option that you might choose either has the difficulties of an attachment with a socket approach or uses the a titanium rod to attach your prosthetic and avoids different negative aspects of a socket attachment device.
		Avoid socket perspiring, skin chafing, and weight	This attachment device is a rod protruding from your bone and avoids the use of a socket and avoids perspiring skin chafing and its heavy weight.	
		Rapid snap on	This attachment devices allows you to rapidly and easily snap your prosthetic to an attachment that protrudes from your bone.	
		Comfort sitting	You are comfortable sitting, including sitting on the toilet with the rod attachment.	
6.	Your sense of limb perception:	No sense of limb connection	You have no sense of limb connection.	Each presented option that you might choose may have a different feeling or sense of connectedness of the prosthetic to your body. This may affect how your motion feels and how well and how much you prefer to use your prosthetic.
		Feels somewhat sense of limb connection	Your prosthetic feels somewhat connected to your body.	
		Feels connected like normal arm	Your prosthetic feels connected like a normal limb on your body.	
7.	Improved motion and fatigue:	Almost normal walking gait	This new attachment device allows you to have almost a normal walking gait.	Each presented option that you might choose emphasizes a different ability to walk or maneuver on different types of ground and with less fatigue with the new prosthetic attachment device.
		Easily maneuver leg into car or under table	This new attachment device allows you to easily maneuver your leg into a car or under a table.	
		Need fewer or no aides on uneven ground	Even when you walk on uneven ground, you have the need for fewer or no aids.	
		Daily, walk longer with less fatigue	Every day you can now walk longer with less fatigue.	
8.	Chance of daily pain:	6 in 10	6 in 10 people will have daily pain or discomfort with this prosthetic attachment device.	Each presented option that you might choose may have a different chance for people to feel daily pain or discomfort due to the attachment device.
		2 in 10	2 in 10 people will have daily pain or discomfort with this prosthetic attachment device.	
		0 in 10	0 in 10 people will have daily pain or discomfort with this prosthetic attachment device.	

### Study Sample

Data collection was from July 2018 to July 2022. The pilot included 73 responses, of which 6 were incomplete and 3 contained incorrect responses to the fixed validity question and thus were eliminated. We then collected an additional 146 survey responses using the same methods, minus 11 incompletes and 11 incorrect responses to the fixed question. We combined these samples for a total analytic sample of 188 individuals with LLL.

### Sample Demographics and Limb-Loss Characteristics

The average age was 49 y (range, 21–88 y). There were slightly more males (56.4%) than females (43.6%), most were married or partnered (57.4%), almost all had obtained a high school diploma or higher (98.9%), and most individuals were employed full-time or retired (62.7%). Most (87.2%) had transtibial unilateral or bilateral amputation (below the knee) and were an average of 35 y old at the time of limb loss. Traumatic accidents were the primary reason for amputation (46.8%), followed by infection and diabetes or loss of circulation (20.7% and 13.8%, respectively). Most individuals were not willing to go without their prosthesis for any period of time, and slightly fewer were willing to go without their prosthesis for 0 to 3 mo only (44.7% and 34.0%, respectively). Rehabilitation time showed 28.7% willing to have 2-h daily sessions for 9 to 12+ mo and 27.7% wanting daily rehabilitation for only 0 to 3 mo (Table 2). Only 3 respondents had an osseointegration attachment device, and only 4 had heard of osseointegration at time of the survey. This is understandable, since the procedure was not yet approved by the FDA at time of the survey.

**Table 2:** Demographic, Limb Loss, and Risk Characteristics.

Characteristic	Study Population		
	Pilot (*n* = 64)	Baseline (*n* = 124)	Total (*N* = 188)
Age, y, $\bar{x}$ (range)	46.7 (21–88)	50.2 (22–86)	49.0 (21–88)
Sex, n (%)			
Male	34 (53.1)	72 (58.1)	106 (56.4)
Female	30 (46.9)	52 (41.9)	82 (43.6)
Transgender/genderqueer/gender nonconforming/other	0 (0)	0 (0)	0 (0)
Marital status, *n* (%)			
Married or partnered	27 (42.2)	81 (65.3)	108 (57.4)
Single	22 (34.4)	29 (23.4)	51 (27.1)
Divorced	11 (17.2)	12 (9.7)	23 (12.2)
Widowed	4 (6.2)	2 (1.6)	6 (3.2)
Education level, *n* (%)			
Less than high school	0 (0)	2 (1.6)	2 (1.1)
High school diploma or equivalent	23 (35.9)	31 (25.0)	54 (28.7)
Undergraduate or college degree	27 (42.2)	54 (43.5)	81 (43.1)
Graduate degree	14 (21.9)	37 (29.8)	51 (27.1)
Employment Status, *n* (%)			
Employed	36 (56.2)	59 (47.6)	95 (50.5)
Unemployed	7 (10.9)	13 (10.5)	20 (10.6)
Retired	12 (18.8)	38 (30.6)	50 (26.6)
Unable to work	9 (14.1)	14 (11.3)	23 (12.2)
Location of limb loss, *n* (%)^a^			
Left below-knee amputation	22 (34.4)	56 (45.2)	78 (41.5)
Left above-knee amputation	8 (12.5)	16 (12.9)	24 (12.8)
Right below-knee amputation	29 (45.3)	57 (46.0)	86 (45.7)
Right above-knee amputation	12 (18.9)	16 (12.9)	28 (14.9)
Age of limb loss, $\bar{x}$ (range)^b^	34.2 (1–73)	35.5 (0–83)	35.1 (0–83)
Reason for limb loss, *n* (%)			
Congenital (present at birth)	3 (4.7)	13 (10.5)	16 (8.5)
Accident	33 (51.6)	55 (44.4)	88 (46.8)
Cancer	12 (18.8)	7 (5.6)	19 (10.1)
Diabetes or loss of circulation	6 (9.4)	20 (16.1)	26 (13.8)
Infection	10 (15.6)	29 (23.4)	39 (20.7)
Willingness to go without lower-limb prosthesis, *n* (%)			
No more than 0–3 mo	25 (39.1)	39 (31.5)	64 (34.0)
No more than 3–6 mo	6 (9.4)	15 (12.1)	21 (11.2)
No more than 6–9 mo	0 (0)	12 (9.7)	12 (6.4)
No more than 9–12 mo	2 (3.1)	5 (4.0)	7 (3.7)
Not willing to go without prosthesis	31 (48.4)	53 (42.7)	84 (44.7)
Willingness to undergo 2 h/wk of rehabilitation, *n* (%)			
No more than 0–3 mo	18 (28.1)	34 (27.4)	52 (27.7)
No more than 3–6 mo	12 (18.8)	27 (21.8)	39 (20.7)
No more than 6–9 mo	6 (9.4)	12 (9.7)	18 (9.6)
No more than 9–12 mo	25 (39.1)	29 (23.4)	54 (28.7)
Not willing to go without prosthesis	3 (4.7)	22 (17.7)	25 (13.3)
Current health status, *n* (%)			
Poor	0 (0)	3 (2.4)	3 (1.6)
Fair	5 (7.8)	27 (21.8)	32 (17.0)
Good	13 (20.3)	39 (31.5)	52 (27.7)
Very good	32 (50.0)	33 (26.6)	65 (34.6)
Excellent	14 (21.9)	22 (17.7)	36 (19.1)
Risk taker, *n* (%)			
No risk	0 (0)	4 (3.2)	4 (2.1)
Mild risk	8 (12.5)	27 (21.8)	35 (18.6)
Moderate risk	25 (39.1)	52 (41.9)	77 (41.0)
Moderately extreme risk	24 (37.5)	33 (26.6)	57 (30.3)
Extreme risk	7 (10.9)	8 (6.5)	15 (8.0)

a Exceeds total of sample. Individuals may have more than 1 limb loss.

b Average for total analytic sample taken from 187 individuals due to missing entry.

### Assessment Scores

The Q-TFA scores, each of which ranged from 0 to 100, demonstrated that our sample at baseline fell about midway between the highest and lowest scores on prosthetic use ( $\bar{x}$ = 52) and use of walking aids ( $\bar{x}$ = 54), with slightly higher than midway scores on capability to use prosthetics in walking ( $\bar{x}$ = 66), overall mobility scores ( $\bar{x}$ = 56), and global function score ( $\bar{x}$ = 64). They also showed a much lower prosthesis problem score ( $\bar{x}$ = 25) than the average score, indicating they had fewer problems with their prosthesis-related mobility (Appendix Table 1).

### Preference Utilities

**Figure 1:**
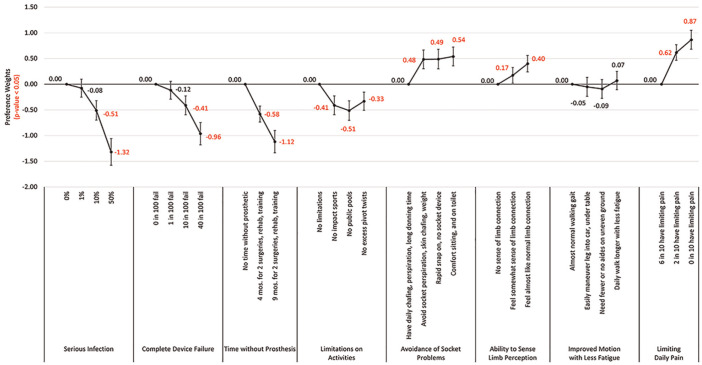
Preference utilities using categorical variables and dummy coding, *N* = 188.

The preference utility β coefficients demonstrated that compared with no risk of serious infection, both avoiding the highest risk (50%) of serious infection (β = −1.32, *P* < 0.001) and moving from avoiding no time without a prosthetic to 9 mo without full prosthetic use for rehabilitation and training (β = −1.12, *P* < 0.001) were the least important levels when compared with other levels of the same attribute (Figure 1 and Appendix Table 2, Appendix Figure 2; now with confidence intervals).

An important positive preference that can be associated with the osseointegration procedure relative to other attributes was the chance to limit daily pain to 0% from a base of 60% having pain (β = 0.87, *P* < 0.001). Other important benefits of the osseointegration procedure relating to its potential to avoid various types of socket problems were increased comfort sitting (β = 0.54, *P* < 0.001), avoidance of limb perspiration and skin chafing (β = 0.48, *P* < 0.001), and the advantage of a rapid device snap on (β = 0.49, *P* < 0.001), compared with a baseline of having daily chafing, perspiration, and long donning times. The importance of these changes in benefits, however, was about half that of the importance of a change from none to the highest infection risk, longest time without prosthetic, and highest change of complete device failure. However, the actual risk of infection associated with osseointegration ranged from 10% to 20% in 10 y. Therefore, the individual’s importance to avoid a 10% chance of infection (compared with no infection risk) is much lower (β = 0.51, *P* < 0.001) than for avoiding the change from no to maximum infection risk, relative to other attributes, and is in line with the importance shown of the benefits of osseointegration.^25^ Individuals were fairly unconcerned about avoiding a 1% risk of infection (compared with no risk) with an importance score of −0.08 relative to other attributes. In addition, the actual risk of complete device failure with osseointegration is about 1.88%, and individuals showed a tolerance for this risk with a preference weight importance of −0.41 for a 10% complete failure rate and −0.12 for a 1% complete failure rate, both when compared with no failure and relative to other attributes (Figure 1, Appendix Table 2).^26^ Therefore, individuals showed they are willing to trade the actual risks for the likely benefits of osseointegration. Avoiding the actual 9-mo loss of the use of their prosthetic during the osseointegration procedure compared with no time without prosthetic use is still strongly important relative to other attributes, however. Compared with no procedure, individuals showed the importance with a preference weight of −0.58 to avoid the 4-mo time frame and much more importance (−1.12) for avoiding the longer 9-mo time frame compared with no time lost and relative to the other attributes. Therefore, if safety considerations are not a factor (as some studies indicate), individuals demonstrate the importance to them of a shorter implant and recovery time.^27^

We also analyzed the base RPL model when including covariates as random effects (dummy coded). Only gender was significant. Being female significantly increased the importance of all the weights in the model.

### Relative Attribute Importance

The importance score demonstrated that avoiding infection risk was the most important attribute, closely followed by avoiding the time without prosthesis due to the osseointegration procedure and the risk of a complete implant failure. Limiting daily pain was the most important benefit attribute, followed by eliminating socket problems and avoiding activity limitations.

**Figure 2:**
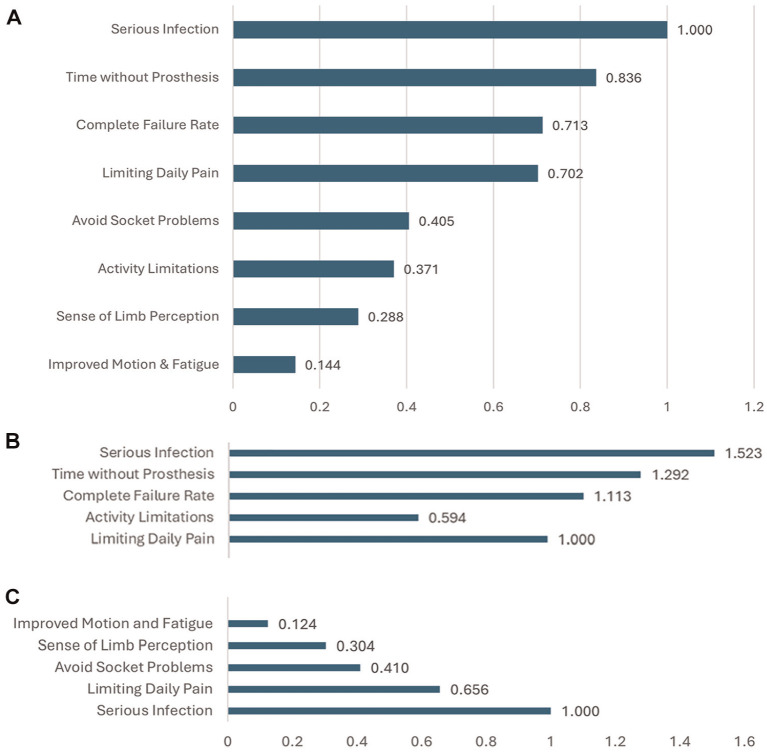
Importance scores: (a) relative attribute level importance score (effects coded), (b) relative attribute maximum acceptable risk (effects coded), and (c) relative attribute minimal acceptable benefit (effects coded).

The relative importance score calculated from effects-coded RPL analysis and then normalized to 1 for serious infection across attributes demonstrated that compared with the importance of avoiding infection risk (the highest utility), avoiding time without the prosthesis was 0.84 as important and avoiding complete failure rate is 0.71 as important. The highest benefit (limiting daily pain) was 0.70 as important as avoiding the infection risk, and avoiding socket problems was 0.40 as important as avoiding infection risk. A sense of limb perception was only 0.29 as important as infection risk (Figure 2a).

When comparing benefits with the most important risks as a continuous variable, a 1% increase in infection risk was determined for a unit improvement in each of the benefits (Figure 2b). We show an average of −0.65 of infection risk for a 1% increase in the chance of avoiding daily pain and a −0.41 of infection risk for a 1-unit improvement in avoiding socket problems. When benefits are compared, we show that a 1.52% chance of daily pain improvement is required for a 1% increase in serious infection risk and a 1.11% chance of daily pain improvement is required for a 1% improvement of risk of complete device failure (almost an equal tradeoff) (Figure 2b, c).

**Figure 3:**
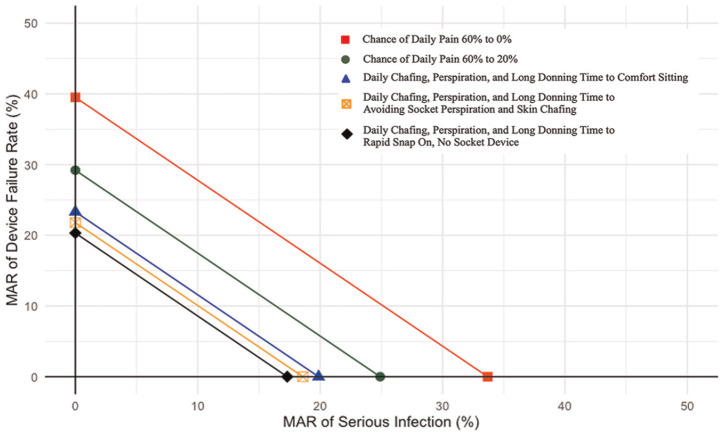
Simultaneous analysis of risks of complete device failure and infection risks across changes in benefits of osseointegration.

To assess the effect of considering the risks and benefits of the MARs simultaneously rather than individually, we used the SMART approach to find the thresholds for a tradeoff of benefits for the 2 most important risks of osseointegration: infection risk and risk of complete device failure. When risks are analyzed as a continuous variable, we show that when moving from a 60% to 0% chance of daily pain, there is an individual MAR of 33.68% for serious infections and 39.52% for complete device failure. Connecting these 2 linearly provides the threshold for the 2 risks considered simultaneously. Any combination of these 2 risks that fall below this threshold are acceptable tradeoffs for a 60% decrease in the chance of daily pain. Figure 3 additionally shows the risk thresholds for moving from a 60% to 20% chance of pain and changes from having socket problems to each improvement level of socket problems. This allows us to assist an individual in assessing important risks simultaneously when making tradeoffs with the benefits of osseointegration.

### Subanalysis

Subanalysis was performed on measures that might affect differences in individual preference for osseointegration, including 1) unilateral or bilateral limb loss, 2) gender, 3) age groups, 4) whether they considered themselves a risk taker or not (scores 1–3), and 5) high versus low Q-TFA scores, including a) prosthetic use score, b) prosthetic mobility, c) problem in life score, and d) global score.

Individuals who had a bilateral limb loss were more risk averse than those with unilateral limb loss for some attributes. The only attribute levels that were significantly different for those with bilateral compared with unilateral LLL were the 2 highest levels of risk of device failure (both compared with no device failure) (level 3: bilateral loss: β = −1.79 v. unilateral loss β = −0.33, *P* = 0.00, and level 4: bilateral loss β = −4.63 v. unilateral loss β = −0.86, *P* = 0.005). Individuals with bilateral loss also showed significant differences in the importance for the highest level of a gain in sense of limb perception (compared with the no limb perception baseline) with bilateral loss (β = −0.23 v. unilateral loss: β = 0.50, *P* = 0.02).

**Figure 4:**
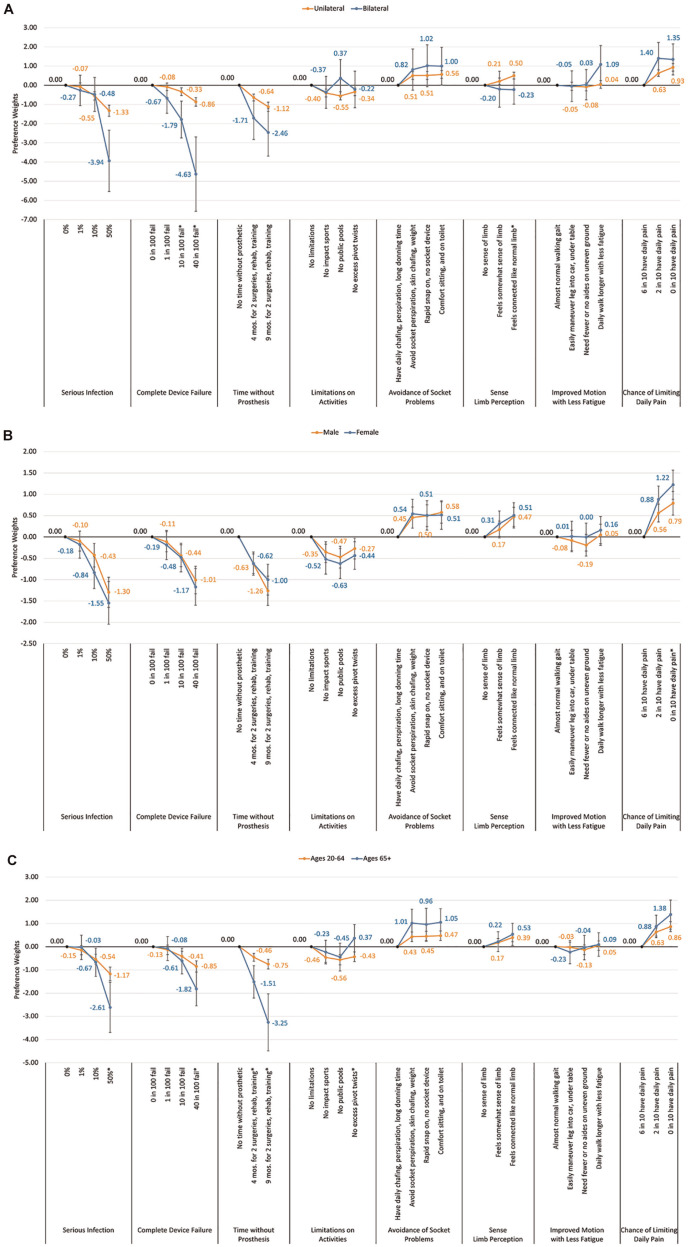
Comparison of preference utiities by individual demographic and limb loss factors: (a) comparison of preference utilities for unilateral vs bilateral lower limb loss, (b) comparison of preference utilities by gender, and (c) comparison of preference utillities by age group.

Importance was also significantly different between men and women but only for the pain avoidance attribute. Women showed significantly more importance to gain the highest daily pain reductions (a change from baseline 60% to 0% chance in daily limiting pain; women: β = 1.22 v. men: β = 0.79, *P* = 0.05; Figure 4b).

There were also significant differences in importance between young (20–64 y) and older (65+ y) age groups. Individuals in the older age group showed significantly more risk aversion to avoid the highest infection risk compared with no infection risk (age 65+ y: β = −2.61 v. age 20–64 y: β = −1.17, *P* = 0.03) and the highest risk of complete device failure compared with none (age 65+ y: β = −1.82 v. age 20–64 y: β = −0.85, *P* = 0.02). The older age group also showed significantly more importance to avoid both 6-mo and 9-mo time frames without prosthetic, required for the surgery and training to have the osseointegration procedure (compared with none) (age 65+ y: β = −1.51 and −3.25 v. age 20–64 y: β = −0.46 and −0.75, *P* = 0.002 and *P* < 0.001, respectively). Some osseointegration procedures limit the age for osseointegration to <65 y, and this may align with their preference to avoid this osseointegration shown here (Figure 4c).

**Figure 5:**
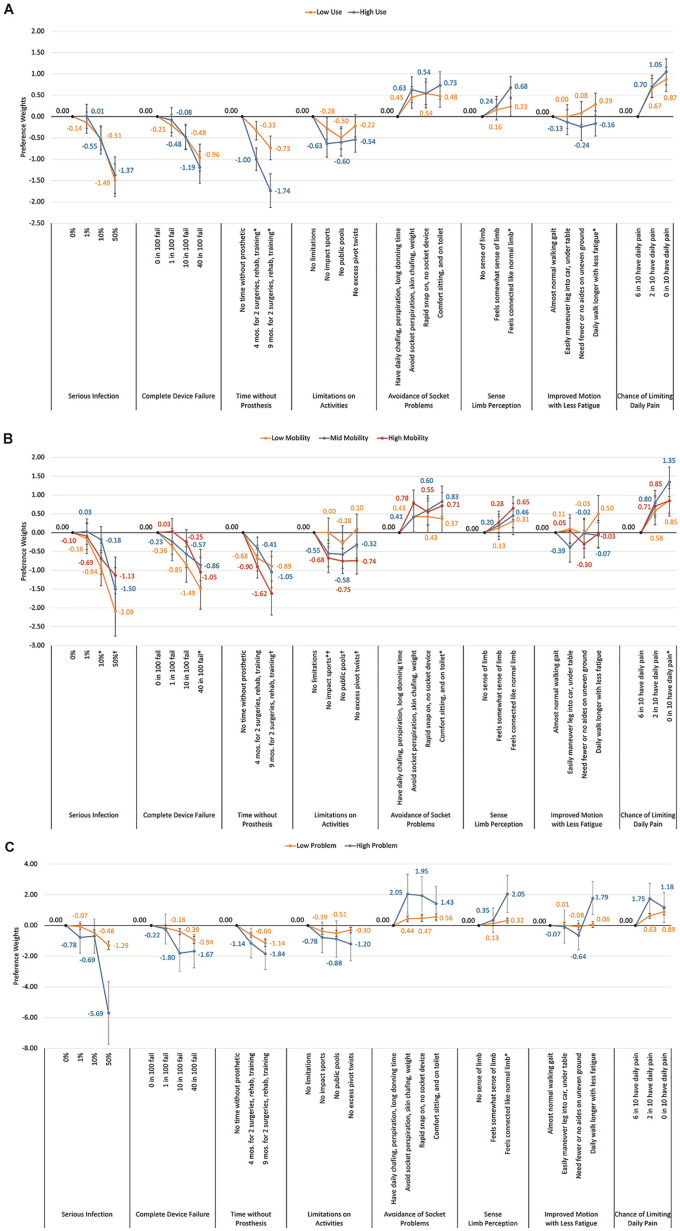
Comparison of preference utilites by Q-TFA scores: (a) by high and low Q-TFA prosthetic use scores, (b) by low mid, and high Q-TFA prosthetic mobility scores, and (c) by low and high Q-TFA prosthetic problem scores.

The subanalysis also showed importance differences in some attributes by Q-TFA scores. Those who scored as a high prosthetic user showed significantly more importance for avoiding both 6 mo (high users: β = −1.00 v. low users β = −0.33, *P* < 0.001) and 9 mo (high users: β = −1.74 v. low users: β = −0.73, *P* < 0.001) without a prosthetic (compared with no time without prosthetic) for the osseointegration procedure and training required. In addition, those with high prosthetic use scores showed significantly more importance for having a normal sense of limb connection compared with no perception of limb connection (high user: β = 0.68 v. low user: β = 0.23, *P* = 0.02). Those with low current prosthetic use scores showed significantly more importance for gaining improved longer daily walking with less fatigue compared with almost normal walking gait (low user: β = 0.29 v. high user: β = −0.16, *P* = 0.03; Figure 5a).

Individuals’ different scores on the Q-TFA functional mobility abilities also showed preference differences. Those with low mobility significantly showed the highest importance for avoiding the highest level of infection risk (50% v. none) when compared with both the groups with middle and highest mobility. The lowest mobility group also showed significantly more importance for avoiding the highest chance (40% compared with no chance) of complete device failure compared with the middle mobility group; the highest mobility group showed significantly more importance for avoiding the longest time (9 mo compared with no time) without prosthetic use when compared with the lowest mobility group.

The group with the highest mobility score also showed significantly more importance to avoid all levels of activity limitations (compared with no activity limitations) as compared with the group with the lowest mobility score. In addition, the high mobility group showed significantly more importance for eliminating socket perspiration and chaffing (compared with baseline daily chafing, perspiration, and long donning time) than the low mobility group did. Both the high and the middle mobility groups showed significantly more importance for experiencing comfort sitting and on the toilet (compared with baseline daily chafing, perspiration, and long donning time) than those with low mobility scores did (main benefits of osseointegration). Finally, those with middle mobility scores compared with those with low mobility scores showed significantly more importance for avoiding the highest level of pain (i.e., moving from 60% to 0% chance of having daily pain) (Figure 5b).

Those experiencing a high level of Q-TFA scored problems with their prosthetic device showed significantly different importance for only 1 attribute level compared with those with low problem scores: a significantly stronger importance for the highest sense of limb perception (almost like a normal limb connection perceived compared with no sense of limb connection perception) (Figure 5c, Appendix Figure 3).

We began to address the possibility of scale differences across subgroups by using the LRT to compare the 6 individual RPL preference models (heteroskedastic) using each subgroup variable as the scale parameter, with the overall base RPL preference model. We found that unilateral versus bilateral loss, gender, and problem score model comparisons were not significant, indicating no likely scale difference across these subgroups. However, the age group (*P* < 0.001), use score (*P* = 0.01), and mobility score comparisons (*P* = 0.01) were all significant, indicating that scale differences are possible across these subgroups. In addition to the analysis run to address scale differences, we also introduced each subanalysis factor (males/females, unilateral v. bilateral limb loss, age group, low/high ease of use) into the RPL to determine the interaction effect and the significance of any difference between each of these groups. We show that only age group exhibited a significant interaction.

### Latent Class Analysis

We modeled 2, 3, 4, and 5 latent classes (dummy coded) and compared them based on the Bayesian information criterion (BIC) and Akaike information criterion (AIC) model fit indices as well as the ability to explain the patterns of the preferences shown in the classes. The lowest BIC and AIC indicated different models as best, with the AIC favoring the model with the best precision fit with 4 classes and the BIC favoring the best model complexity with 2 classes (Table 3).

**Table 3:** Latent Class Model Differentiation Statistics

Dummy Coded				Probability of Belonging in Each Class				
Model	Log-Likelihood	AIC	BIC	1	2	3	4	5
2 Class	−1,926.13	3,938.26	4,201.72	0.1665	0.8335	—	—	—
3 Class	−1,876.01	3,882.02	4,280.27	0.1576	0.6862	0.1562	—	—
4 Class	−1,817.52	3,809.04	4,342.07	0.1963	0.4316	0.1151	0.2570	—
5 Class	−1,783.39	3,784.78	4,452.60	0.2067	0.0974	0.1169	0.3251	0.2540
Effects Coded				Probability of Belonging in Each Class				
Model	Log-Likelihood	AIC	BIC	1	2	3	4	5
2 Class	−1,926.13	3,938.26	4,201.71	0.8342	0.1658	—	—	—
3 Class	−1,868.09	3,866.17	4,264.42	0.5608	0.2594	0.1798	—	—
4 Class	−1,817.52	3,809.04	4,342.07	0.4316	0.1152	0.2568	0.1963	—
5 Class	−1,784.14	3,786.28	4,454.10	0.0562	0.4222	0.1131	0.2425	0.1660

AIC, Akaike information criterion; BIC, Bayesian information criterion.

**Figure 6:**
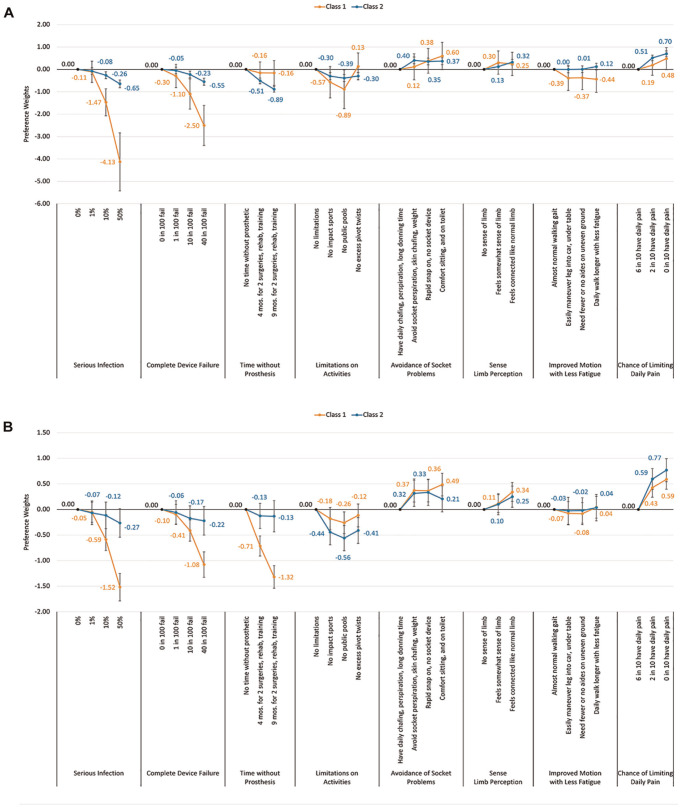
Patient heterogeneity across two-class latent class analysis: risk averse and mixed response groups. (a) two-class latent class analysis with no covariates, and dummy coded, and (b) two-class latent class analysis with covariates (age group, gender, unilateral/bilateral loss, Q-TAF use score) and dummy coded.

We present the 2-class model with class 1 showing the strong importance for risk avoidance (infection and failure rate risks) and also a strong preference for most of the benefits afforded by the osseointegration implant (avoidance of daily pain, improved mobility with less fatigue, and avoidance of chafing and perspiration with a socket). There was a 17% probability of being in class 1. The 83% in class 2 showed more of a moderate level of importance for both risk avoidance and benefit seeking, although they showed a much stronger importance for avoiding the time without a prosthetic required for osseointegration. These results indicate that those falling in either class might require much more individualized discussion about how much and what type of risk and benefit they are willing to trade to help in osseointegration decision making. The 3-class model showed similar, but more moderate, groups compared with the class 2 model, except those in class 3 showed an extremely high importance for gaining a decrease in the chance of having daily pain. This could be an important distinct group, which is having significant pain, and would need consultation to determine if osseointegration surgery could afford the added benefit of diminishing their specific pain problem (Figure 6a and b, Appendix Figure 4).

We also ran the 2-group latent class model with covariates as interaction terms to determine significant differences in group membership when these were considered. We showed that males and those with higher Q-TFA prosthetic user scores were more likely to belong in group 1, while those aged 65+ y and those with bilateral limb loss were more likely to belong in group 2.

## Discussion

This study is the first to examine discrete-choice preferences for the risks and benefits when making a decision as to whether to adopt an osseointegration attachment device. We demonstrate that although a strong importance is shown for avoiding the risk of infection and the highest risks of complete device failure, as well as the time without a prosthetic during surgery, training, and rehabilitation required with osseointegration implantation, individuals are also willing to trade these risks at the current device levels for the expected benefits of osseointegration attachment. The actual risk of infection associated with osseointegration ranges from 10% to 20% in 10 y and about 9% in those using the US-approved osseointegration system.^28^ Our preference data indicate that the importance shown to avoid a 10% chance of infection is about the same (β = −0.51, *P* < 0.001) as the importance shown for the benefits that can be gained from osseointegration (avoiding socket problems [β = 0.485, 0.49, and 0.54] and reduction in daily pain [β = 0.88]).^18,25^ These results show that the importance shown to avoid the actual infection risk can be offset by the preference for the benefits of osseointegration to avoid pain and socket problems and to have rapid device snap on. In addition, the actual risk of complete device failure with osseointegration is about 1.88%, and individuals showed a tolerance for this risk with a negative importance weight of −0.42 for a 10% complete failure rate and −0.12 for a 1% complete failure rate compared with no failure.^26^ Therefore, individuals also showed they are willing to trade the actual failure rate risks for the likely benefits of osseointegration.

Avoiding time without the use of a prosthetic for the time it takes to undergo and recover from the osseointegration procedure is also a strongly negatively preferred risk. There is considerable debate currently about whether a 1- or 2-stage osseointegration procedure is safer, with the 1-stage procedure providing a faster recovery time. Our attribute that compares the loss of prosthesis use for either a 4-mo versus a 9-mo time for surgery and rehabilitation to none gives some insight into individuals’ preferences for the time it takes before an expected return to full prosthetic use. Compared with no procedure, individuals have a −0.58 preference to avoid the 4-mo recovery time frame and almost the same (0.54) additional negative preference for the longer 9-mo time frame. Therefore, if safety considerations are not a factor (as some studies indicate), patients prefer a shorter implant and recovery time.^27^ However, again, avoiding this time without prosthesis use is still in the range of the benefits of osseointegration, showing a willingness to make risk–benefit tradeoffs here.

Individuals showed the strongest importance for a potential benefit of osseointegration to reduce the presence of daily pain. Pain is often caused by a poorly tolerated prosthetic socket, and osseointegration can avoid these difficulties completely. In addition, however, the osseointegration procedure is often accompanied by a targeted muscle reinnervation (TMR) procedure to alleviate pain and prevent future neuroma development. One study of 100 patients with osseointegration demonstrated a reduction in pain with osseointegration both with and without the TMR procedure. Those who developed new neuromas showed unchanged pain scores.^29^ This suggests that pain reduction is a likely benefit of osseointegration, especially when accompanied by TMR, and our study shows this reduction in pain is strongly desired by individuals with LLL.

We showed considerable heterogeneity across patients in their pattern of preferences for the risks and benefits of osseointegration for some levels of some attributes. There is clearly a group of individuals who strongly want to avoid any risk of infection and complete device failure and are not willing to trade these risks for the level of the benefits that they also prefer. While this is a smaller group of individuals (17%) in our sample, it is an important group to identify when discussing this procedure. Subanalysis showed that those who currently show high use and mobility on the Q-TFA measure have a lower threshold for preferences to trade risks that may compromise their current functional abilities (time without prosthetic for procedure and risk for complete device failure) for their perceived benefits. This is perfectly understandable, as they likely are also not having socket or pain problems that can highly benefit those switching to an osseointegrated anchoring device. Currently, the criteria for osseointegration eligibility address some of these concerns, such as a requirement for current difficulties with or the inability to use a current conventional socket prosthesis. Our results show that those whose Q-TFA problem score suggests that they are having problems with their current prosthetic have a much higher preference for all the benefit attributes than those with low problem scores do. Interestingly, however, those with high problem scores are also much more risk averse for all the osseointegration attributes of risk.

Inclusion criteria in the United States also suggest that individuals must be older than 22 y and younger than 65 y to ensure strong bone for the implant.^30,31^ Our subanalysis demonstrating patient preference for those 65+ y demonstrated that although they strongly prefer the benefits osseointegration can provide, they also show the strongest preference for avoiding the risks associated with implantation and the use of osseointegration; thus, as a group, they may not be willing to make this tradeoff of risks for benefits, anyway. These DCE preference results provide population-based preferences and are valuable for informing those regulatory agencies making approval decisions as well as for use in clinical discussions between patient and clinician when making decisions about attachment device options. These results should accompany more specific individualized discussions to assist in clinical decision making to best improve patient outcomes.

A potential weakness of DCE studies is the presence of potential scale differences, especially when comparing across subgroups as we do. We began to address the possibility of scale differences across subgroups by using the LRT to compare the 6 individual RPL preference models (heteroskedastic) using each subgroup variable as the scale parameter, with the overall base RPL preference model. We found that the age group comparison was significant (*P* < 0.001) and the use score (*P* = 0.011) and mobility score comparisons (*P* = 0.007) were also significant, indicating that scale differences are possible across these subgroups. We also showed that age group exhibited significant interactions in our RPL analysis across subgroups. Therefore, we cannot rule out that the error variances accounted for preference weight differences shown in our analysis for these subgroup comparisons. Another potential weakness is the possibility of nonattendance by respondents.^32^ We used several methods to address another inherent weakness of CBC models, which is to correct for respondents’ low-quality response patterns. Our preference comparisons primarily account for task nonattendance by using a fixed question comparing all the best and all the worst levels of the attributes for the paired choice. Those who chose all the worst choices were eliminated. We also removed anyone who was straight lining. We also had a more “hands-on” approach to recruitment than many other studies have had, often meeting the respondents in person to ensure their eligibility and interest. Nonetheless, this does not preclude nonattendance, and of course, this could differ between our subgroups and be a weakness of our results. We did ask how confident our respondents were in their responses, and on a 1- to 10-point Likert-type scale, only 3 respondents indicated a confidence less than 5 and 7 individuals less than 6. However, this was too small a sample to compare preferences across low and high confidence levels to further support the quality of our sample responses. In addition, we addressed low-quality responses with our garbage class MIXL analyses, which, when compared with the standard class MIXL analysis (both conducted in STATA), showed that the size of the garbage class in our dataset was approximately 33% (Appendix Table 3).^24^ In the standard panel MIXL model, the achieved MALA acceptance rates ranged from 0.67 to 0.71, which is close to the target rate of 0.69. It is also reassuring that the Monte Carlo standard errors expressed as a percentage of the SDs of the posterior samples were smaller than 5% to 10% and so are accurately approximated.^24^ The respondents preferred most to avoid time without a prosthetic and then serious infection, chance of daily pain, and complete device failure. There was also good variation in respondent-specific preferences, as the SD estimates were about as large as the mean estimates were^24^ (Appendix Table 3). The means from the garbage MIXL model showed estimates that were corrected for the those with poor-quality data. Although these garbage-corrected results showed stronger mean estimates, the order of the preferences remained the same, despite an estimated garbage class of 33% (Appendix Table 3). Here, the achieved MALA acceptance rates were still close to the target rates. In addition, the SDs remained between the goal of 5% and 10%. This consistency across these 2 models gives some assurance in the accuracy of the level of preferences in our results.

## Conclusion

In December 2020, the US FDA approved the Osseoanchored Prostheses for Rehabilitation of Amputees (OPRA) implant system in the United States for adults with transfemoral amputations who have difficulty with or cannot use a conventional socket prosthesis attachment, although the OPRA system has been on the market since 2015 under a humanitarian device exemption.^33,34^ This approval has substantially increased the number of osseointegration procedures performed in the United States while other countries are also implanting different varieties of osseointegrated devices as well.^35^ Therefore, as we have done here, the identification of patient preferences for the risk and benefit tradeoffs inherent in the decision making for adopting an osseointegration anchoring device is timely. We demonstrated that individuals with LLL are willing to trade the current risk levels of osseointegration for the likely benefits that this implant can provide. We also demonstrated a heterogeneity of preferences across different patient subgroups that should be kept in mind in patient–clinician discussions during the decision-making process for attachment devices.

## Supplemental Material

sj-docx-1-mpp-10.1177_23814683251351044 – Supplemental material for Preferences for Attachment Devices for Individuals with Lower-Limb Loss: A Discrete-Choice Study to Inform Regulatory Decisions Supplemental material, sj-docx-1-mpp-10.1177_23814683251351044 for Preferences for Attachment Devices for Individuals with Lower-Limb Loss: A Discrete-Choice Study to Inform Regulatory Decisions by Leslie Wilson, Matthew Garibaldi, Ruben Vargas and Molly Timmerman in MDM Policy & Practice

sj-docx-2-mpp-10.1177_23814683251351044 – Supplemental material for Preferences for Attachment Devices for Individuals with Lower-Limb Loss: A Discrete-Choice Study to Inform Regulatory Decisions Supplemental material, sj-docx-2-mpp-10.1177_23814683251351044 for Preferences for Attachment Devices for Individuals with Lower-Limb Loss: A Discrete-Choice Study to Inform Regulatory Decisions by Leslie Wilson, Matthew Garibaldi, Ruben Vargas and Molly Timmerman in MDM Policy & Practice
