# Supplementary material for: Preferences for Attachment Devices for Individuals with Lower-Limb Loss: A Discrete-Choice Study to Inform Regulatory Decisions
Source: MDM Policy Pract. 2025 Jul 2;10(2):23814683251351044. doi: 10.1177/23814683251351044 (PMC12231977; doi:10.1177/23814683251351044)

**Appendices**

**Appendix Table 1: Q-TFA Prosthetic Use, Mobility, Problem and Global Scores**

| Variable | Prosthesis Use | Prosthesis Mobility | | | | Problem | Global |
| --- | --- | --- | --- | --- | --- | --- | --- |
|  |  | Sub Scores | | | Overall |  |  |
|  |  | Walking Aids | Capability | Walking Habits |  |  |  |
| Mean | 52 | 54 | 66 | 47 | 56 | 25 | 64 |
| Median | 51 | 50 | 67 | 45 | 55 | 20 | 58 |
| Standard Deviation | 26 | 40 | 28 | 32 | 29 | 21 | 22 |
| Minimum | 0 | 0 | 0 | 0 | 0 | 0 | 0 |
| Maximum | 100 | 100 | 100 | 100 | 100 | 87 | 100 |

Note: All separate scores range from 0-100.

Prosthetic Use: high score means high use so high scores are better

Prosthetic Mobility Subscores = Walking Aids: high score is better as it means no or fewer walking aids, Capability: high score is better because it means they are more capable of using prosthetics and walking, Walking Habits: higher scores mean a better walking capability so a high score is better, Overall Score: means better mobility so a higher score is better.

Problem: high score means that there are more problems so a high score is worse and a low score is better as it means less problems.

Global: high score means there is better function, fewer problems and a better overall situation as an amputee, so a high score is better.

**Appendix Table 2: Preference Utilities using categorical variables and dummy coding**

**Appendix Figure 1: Example of Choice Pair Question from the DCE CBC measure:**


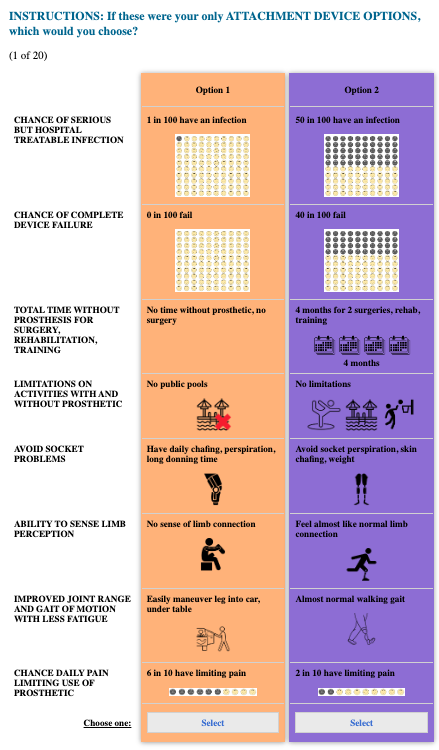


**Appendix Figure 2: Preference weights (utilities) n=188 (mixed logit using continuous variables and dummy coding**

Note: mixed logit analysis using continuous variables and dummy coding


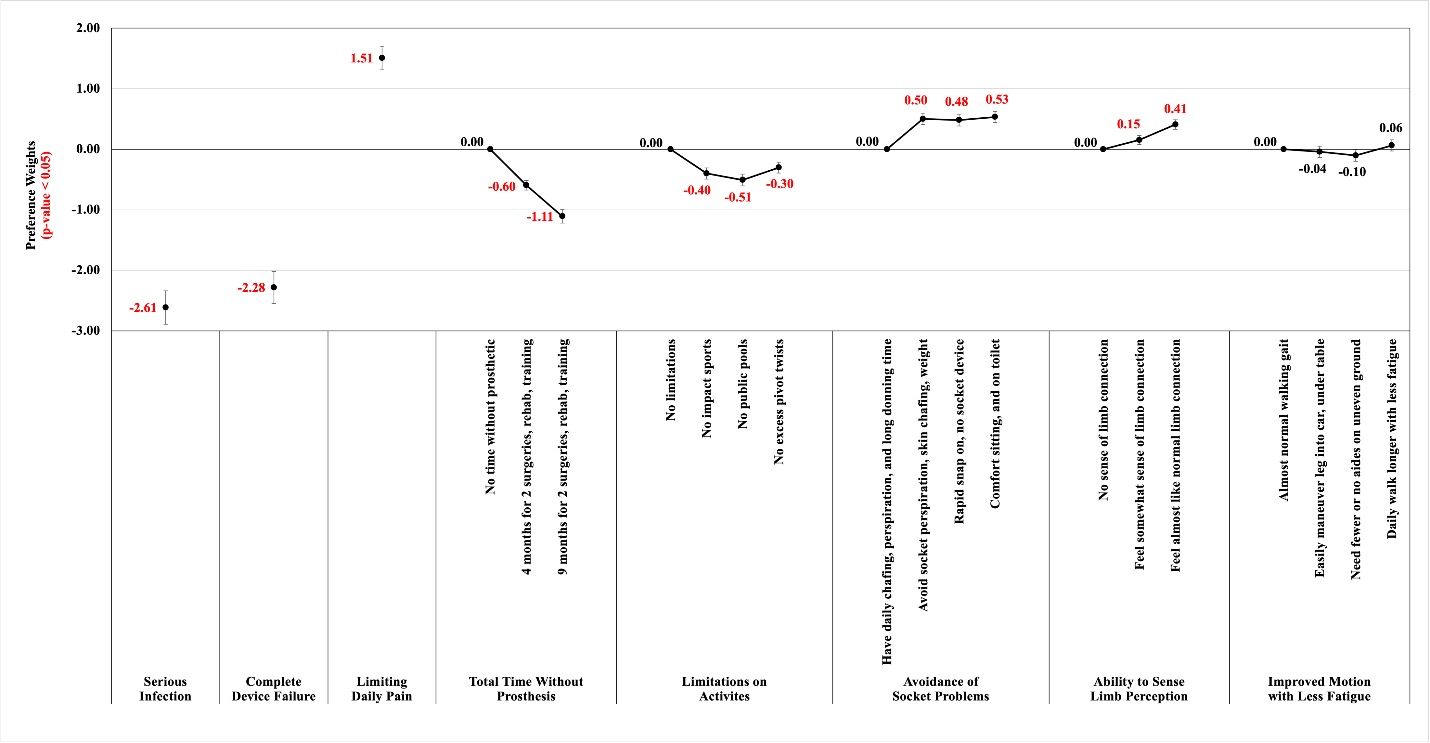


**Appendix Figure 3: Comparison of Preference Utilities by Q-TFA Scores, Low, Mid, and High Q-TFA Prosthetic Global Scores)**


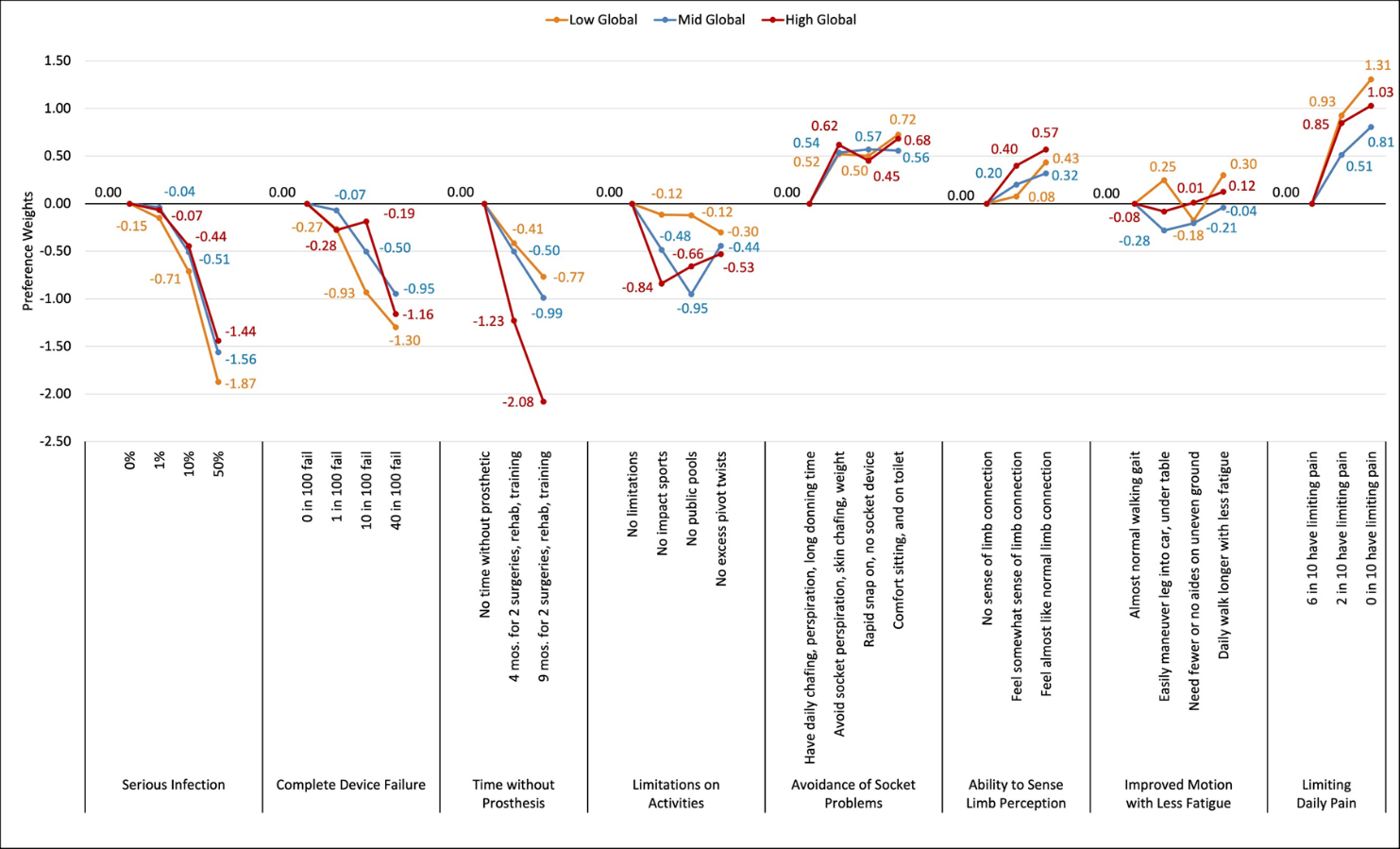


**Appendix Figure 4: Three-Class Latent Class Analysis**


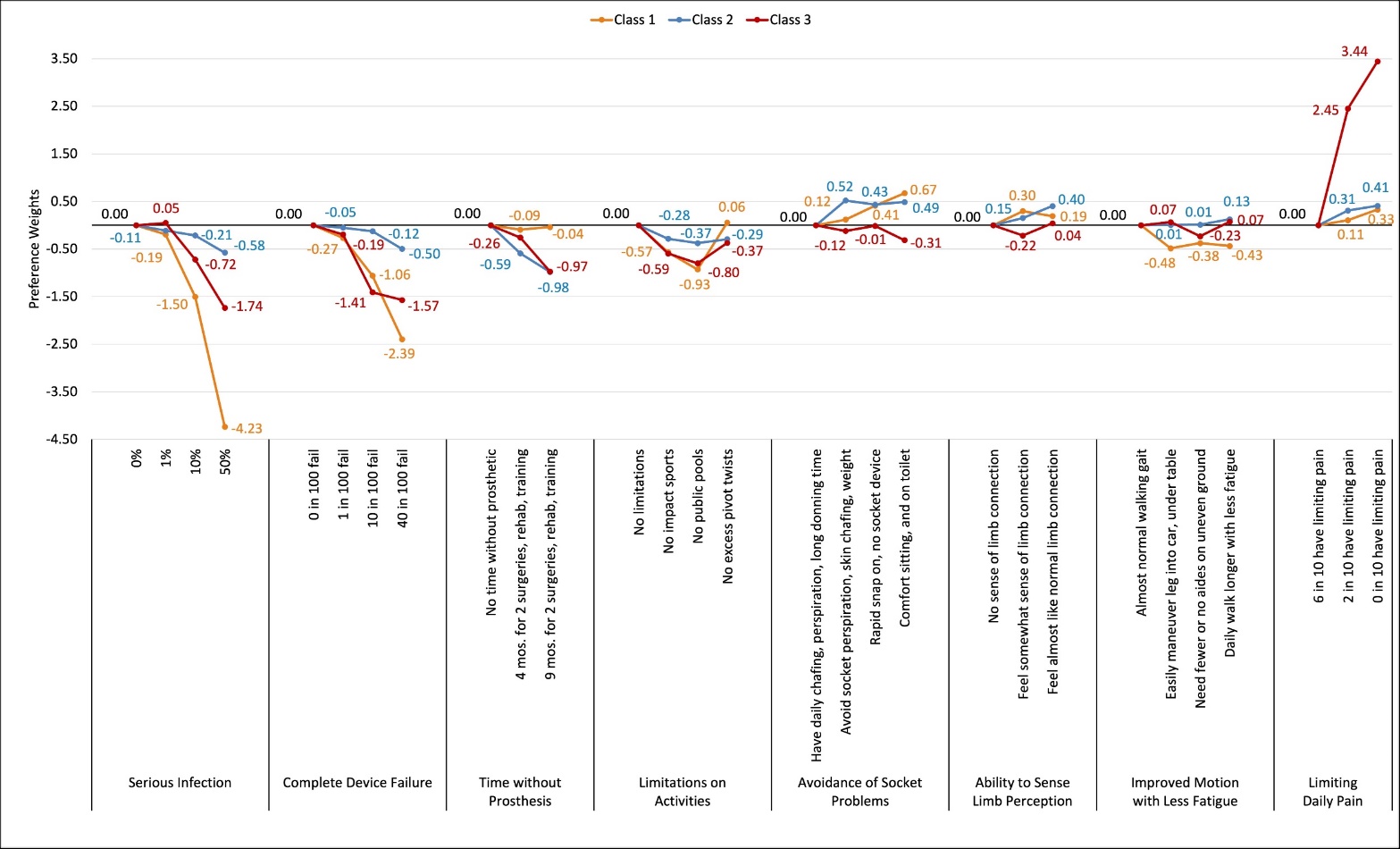

Supplement: sj-docx-1-mpp-10.1177_23814683251351044 – Supplemental material for Preferences for Attachment Devices for Individuals with Lower-Limb Loss: A Discrete-Choice Study to Inform Regulatory Decisions [file sj-docx-1-mpp-10.1177_23814683251351044.docx]
