# Supplementary material for: Preferences for Attachment Devices for Individuals with Lower-Limb Loss: A Discrete-Choice Study to Inform Regulatory Decisions
Source: MDM Policy Pract. 2025 Jul 2;10(2):23814683251351044. doi: 10.1177/23814683251351044 (PMC12231977; doi:10.1177/23814683251351044)
Supplement: Supplementary file 2 [file sj-docx-2-mpp-10.1177_23814683251351044.docx]

Table 3 Appendix: Garbage Class Estimates compared with Standard Mixed effects Means

**Standard Bayesian MIXL Estimates**				**Bayesian Garbage Class MIXL Estimates**			
Mean							
**Attributes**	**Mean**	**Std. Dev.**	**MCSE**	**Attributes**	**Mean**	**Std. Dev.**	**MCSE**
Serious Infection	-0.427797	0.043268	0.001214	Serious Infection	-0.737965	0.078857	0.006235
Complete Failure Rate	-0.332004	0.038193	0.001396	Complete Failure Rate	-0.564457	0.064261	0.005196
Time without Prosthetic	-0.532185	0.059479	0.001563	Time without Prosthetic	-0.916407	0.121034	0.008642
Activity Limitations	-0.098310	0.028890	0.001786	Activity Limitations	-0.126904	0.048429	0.002442
Avoid Socket Problems	0.162530	0.028752	0.001765	Avoid Socket Problems	0.241506	0.051738	0.002720
Sense Limb Perception	0.194042	0.041762	0.001920	Sense Limb Perception	0.285448	0.069935	0.003952
Improved Motion & Fatigue	0.030255	0.029909	0.001885	Improved Motion & Fatigue	0.041646	0.042094	0.004406
Chance of Daily Pain	0.423589	0.054430	0.001667	Chance of Daily Pain	0.694757	0.102719	0.005736
**SD**				**SD**			
	**Mean**	**Std. Dev.**	**MCSE**		**Mean**	**Std. Dev.**	**MCSE**
Serious Infection	0.410266	0.0463	0.00189	Serious Infection	0.48928	0.08129	0.00402
Complete Failure Rate	0.315903	0.04477	0.00271	Complete Failure Rate	0.37699	0.08107	0.00628
Time without Prosthetic	0.609293	0.064	0.00258	Time without Prosthetic	0.91949	0.12503	0.01158
Activity Limitations	0.130177	0.04524	0.0049	Activity Limitations	0.22164	0.08004	0.00877
Avoid Socket Problems	0.145519	0.05096	0.00743	Avoid Socket Problems	0.266	0.08459	0.0094
Sense Limb Perception	0.256218	0.06203	0.00606	Sense Limb Perception	0.39779	0.11415	0.00797
Improved Motion & Fatigue	0.10908	0.05012	0.00768	Improved Motion & Fatigue	0.11671	0.06215	0.00833
Chance of Daily Pain	0.516051	0.06023	0.00316	Chance of Daily Pain	0.76109	0.1113	0.0098
				**Garbage Class Share**	0.330686	0.021818	0.001323
MALA acceptance rates:				MALA acceptance rates			
Min = 0.67, ave = 0.69, max - 0.71				garbage class = 0.57			
				MIXL class: min = 0.56, Ave = 0.59, max = 0.61			

	**Table 1 Supplement: Garbage Class Estimates compared with Standard Mixed effects Means**							
**Standard Bayesian MIXL Estimates**					**Bayesian Garbage Class MIXL Estimates**			
**Attributes**	**Mean**	**Std. Dev.**	**MCSE**		**Attributes**	**Mean**	**Std. Dev.**	**MCSE**
Serious Infection	-0.427797	0.043268	0.001214		Serious Infection	-0.737965	0.078857	0.006235
Complete Failure Rate	-0.332004	0.038193	0.001396		Complete Failure Rate	-0.564457	0.064261	0.005196
Time without Prosthetic	-0.532185	0.059479	0.001563		Time without Prosthetic	-0.916407	0.121034	0.008642
Activity Limitations	-0.098310	0.028890	0.001786		Activity Limitations	-0.126904	0.048429	0.002442
Avoid Socket Problems	0.162530	0.028752	0.001765		Avoid Socket Problems	0.241506	0.051738	0.002720
Sense Limb Perception	0.194042	0.041762	0.001920		Sense Limb Perception	0.285448	0.069935	0.003952
Improved Motion & Fatigue	0.030255	0.029909	0.001885		Improved Motion & Fatigue	0.041646	0.042094	0.004406
Chance of Daily Pain	0.423589	0.054430	0.001667		Chance of Daily Pain	0.694757	0.102719	0.005736
					**Garbage Class Share**	0.330686	0.021818	0.001323

	Table 1 Supplement: Garbage Class Estimates compared with Standard Mixed effects Mea							
**Standard Bayesian MIXL Estimates**					**Garbage Class MIXL Estimates**			
**Attributes**	**Mean**	**Std. Dev.**	**MCSE**		**Attributes**	**Coef**	**Std. Dev.**	**MCSE**
Serious Infection	-0.427797	0.043268	0.001214		Serious Infection	-0.73797	0.078857	0.006235
Complete Failure	-0.332004	0.038193	0.001396		Complete Failure	-0.56446	0.064261	0.005196
Time without Pros	-0.532185	0.059479	0.001563		Time without Prost	-0.91641	0.121034	0.008642
Activity Limitation	-0.098310	0.028890	0.001786		Activity Limitations	-0.1269	0.048429	0.002442
Avoid Socket Prob	0.162530	0.028752	0.001765		Avoid Socket Probl	0.241506	0.051738	0.002720
Sense Limb Perce	0.194042	0.041762	0.001920		Sense Limb Perce	0.285448	0.069935	0.003952
Improved Motion	0.030255	0.029909	0.001885		Improved Motion 8	0.041646	0.042094	0.004406
Chance of Daily P	0.423589	0.054430	0.001667		Chance of Daily Pa	0.694757	0.102719	0.005736
					**Garbage Class Sh**	0.330686	0.021818	0.001323
